# Effects of future climate on coral-coral competition

**DOI:** 10.1371/journal.pone.0235465

**Published:** 2020-08-13

**Authors:** Nicole K. Johnston, Justin E. Campbell, Valerie J. Paul, Mark E. Hay

**Affiliations:** 1 School of Biological Sciences and Aquatic Chemical Ecology Center, Georgia Institute of Technology, Atlanta, GA, United States of America; 2 Department of Biological Sciences, Institute of Environment, Florida International University, North Miami, FL, United States of America; 3 Smithsonian Marine Station, Ft. Pierce, FL, United States of America; Helmholtz-Zentrum fur Ozeanforschung Kiel, GERMANY

## Abstract

As carbon dioxide (CO_2_) levels increase, coral reefs and other marine systems will be affected by the joint stressors of ocean acidification (OA) and warming. The effects of these two stressors on coral physiology are relatively well studied, but their impact on biotic interactions between corals are poorly understood. While coral-coral interactions are less common on modern reefs, it is important to document the nature of these interactions to better inform restoration strategies in the face of climate change. Using a mesocosm study, we evaluated whether the combined effects of ocean acidification and warming alter the competitive interactions between the common coral *Porites astreoides* and two other mounding corals (*Montastraea cavernosa* or *Orbicella faveolata*) common in the Caribbean. After 7 days of direct contact, *P*. *astreoides* suppressed the photosynthetic potential of *M*. *cavernosa* by 100% in areas of contact under both present (~28.5°C and ~400 μatm *p*CO_2_) and predicted future (~30.0°C and ~1000 μatm *p*CO_2_) conditions. In contrast, under present conditions *M*. *cavernosa* reduced the photosynthetic potential of *P*. *astreoides* by only 38% in areas of contact, while under future conditions reduction was 100%. A similar pattern occurred between *P*. *astreoides* and *O*. *faveolata* at day 7 post contact, but by day 14, each coral had reduced the photosynthetic potential of the other by 100% at the point of contact, and *O*. *faveolata* was generating larger lesions on *P*. *astreoides* than the reverse. In the absence of competition, OA and warming did not affect the photosynthetic potential of any coral. These results suggest that OA and warming can alter the severity of initial coral-coral interactions, with potential cascading effects due to corals serving as foundation species on coral reefs.

## Introduction

Though coral reefs cover <0.1% of the Earth’s surface [1], they are among Earth’s most economically and ecologically valuable ecosystems [2]. In recent decades, coral reefs have been negatively impacted by a wide range of anthropogenic influences such as eutrophication, overfishing, and climate change [3–5] that have led to a 50–80% decline in global coral cover [6, 7]. Ocean acidification (OA) and elevated temperatures are exacerbating this decline [4, 5]. Over the next 100 years, average sea surface temperatures are expected to increase 1–2°C, and ocean pH levels are predicted to decrease by 0.3–0.5 units under the IPCC “business as usual” scenario [8, 9]. The 2015 and 2016 mass bleaching events in the tropics brought on by higher than average water temperatures demonstrated that the effects of climate change are already dramatically and rapidly impacting coral reefs [4], with a recent, five-fold increase in the frequency of mass bleaching events [10]. These rapid changes emphasize the need to better understand and predict the effects of climate change on these valued systems.

Given the foundational role of corals in tropical reef ecosystems, the effects of OA and/or warming on coral physiology have been commonly investigated [11]. Numerous previous studies have focused solely on the effects of either OA or warming on species physiology and interactions with fewer studies focused on the combined effects of OA and warming even though they will be joint stressors [12]. OA reduces calcification and growth rates [13, 14], causes expulsion of dinoflagellate endosymbionts (Symbiodiniaceae) [15], and, in worst cases, leads to coral death [16, 17]. At the same time, the magnitude of the physiological responses to OA and warming can vary among taxonomic groups, populations, and locations [15, 18–21]. Some populations of certain species, such as the coral *Porites astreoides*, have been shown to be able to withstand environmental stressors [22]. Further, meta-analysis and modeling studies have found that responses to OA and warming vary among taxonomic groups and species [21, 23]. These complicated, context-dependent responses to OA and warming make predicting the potential outcomes of interactions between species difficult and highlight the need for direct investigations of how OA and warming impact coral-coral interactions [23].

Corals compete for space on coral reefs with many competitors including algae [24], sponges [25, 26], soft corals [27], ascidians [28], and other hard corals [29–31]. Of these interactions, the effects of OA and warming on coral-coral interactions have rarely been investigated. This may be due to declines in coral cover and increases in algal cover that have led to an increase in coral-algal interactions on many reefs [12, 32]. However, regions or habitat patches with high coral cover can still be found across the globe [33]. Moreover, given the right conditions, some reefs have been shown to rapidly recover from low to high coral cover [34, 35]. These results demonstrate that in certain locations, given proper management strategies, coral-coral interactions are not uncommon.

Corals compete via numerous mechanisms, but in the short-term, competition is commonly via mesenterial filaments used to digest neighboring corals’ tissues [29, 30]. In some cases, initial interactions can reverse when losing colonies form larger sweeper tentacles in areas of contact and reverse the competitive interaction [36, 37]. Coral communities can be shaped by competitive hierarchies [29, 30], with uninterrupted competition leading to changes in coral diversity [38]. A limited number of studies focused on understanding the effects of OA on coral-coral interactions have found mixed results. In some cases, OA affected coral growth (i.e. linear extension) without affecting competition [39] or led to the early extrusion of mesenterial filaments [40]. In another case, OA significantly impacted intraspecific competitive interactions in five species while leaving strong interspecific competitive interactions unaffected. A follow-up model suggested that these changes in competition can shift competitive hierarchies and lower overall coral cover [41]. These studies indicate that the effects of OA on coral-coral competition are mixed and suggest the need for further study. Moreover, the simultaneous effects of increased carbon dioxide and temperature on coral-coral competition have not been evaluated.

This study assessed how the combined stressors of OA and warming affect competition among common mounding corals in the Caribbean (*P*. *astreoides* versus *O*. *faveolata* and *M*. *cavernosa*). These species are among the most common corals in the Florida Keys, are considered to be relatively resistant to several common stresses (e.g. bleaching, predation [42]), and may be the most common remaining competitors on modern, and likely future, Caribbean reefs.

## Materials and methods

### Ethics statement

Field research was conducted under FKNMS-2015-078-A1 and FKNMS-2017-128 research permits granted through the Florida Keys National Marine Sanctuary.

Experiments assessing the combined effects of OA and warming on coral-coral competition were conducted at the Smithsonian Marine Station in Fort Pierce, Florida. Two experiments were run sequentially over a period of seven weeks, with the first experiment running for 16 days between 2 and 18 October 2017 and the second experiment running for 23 days between 18 October and 9 November 2017. Three colonies of *M*. *cavernosa* and *O*. *faveolata*, and six colonies of *P*. *astreoides* (each 30–45 cm in diameter) were collected from the Florida Keys National Marine Sanctuary coral nursery in Key West, FL, maintained in indoor raceways with running seawater for at least 2 months, and then used in the experiments.

### Experiment one: *Porites astreoides* vs *Montastraea cavernosa*

After the initial two-month acclimation period, three colonies of *P*. *astreoides* and three of *M*. *cavernosa* were each divided into eight fragments (each fragment 7–10 cm). Coral fragments recovered in the raceways for 48 hours prior to being moved to 12 tanks of 37-L designed to manipulate carbon dioxide levels and temperature individually in each tank. This short recovery period (48 hours) may have led to some additional coral stress, but it was consistent across all treatments and no apparent signs of stress such as tissue loss or bleaching were observed.

Seawater was collected from 0.4 km offshore from Fort Pierce, Florida, filtered (<10 μm), and water recirculated within each tank using a 473 LPH powerhead. Tanks were randomly assigned to either present (targeted to be ~400 μatm *p*CO_2_ and ~28.5° C) or future (targeted to be ~1000 μatm *p*CO_2_ and ~30.0° C) oceanic conditions (Table 1, n = 6 per treatment). Present treatments are based on current conditions, while the future treatments were designed to mimic the predicted most extreme scenario by the IPCC (RCP 8.5) ([43]) (Table 1). This design was chosen over a fully factorial experiment because temperature and OA are predicted to both change over time, thus representing future conditions, and to allow for greater sample size and power to detect effects on coral-coral interactions. pH was continuously controlled and monitored using a pH stat computer (Aqua Medic) that bubbled 100% CO_2_ into each tank (25 mL/min) as necessary to maintain treatment levels. Temperature was monitored and controlled using independent dual-stage digital controllers attached to water-jacketed heat exchangers. pH, temperature, and salinity were also externally monitored and verified daily using a ThermoFisher Orion Star pH meter (relative accuracy ±0.01 units) and a YSI temperature/salinity meter. Water changes (25%) occurred twice weekly with small additions of deionized (DI) water daily as needed to maintain salinity near 36ppt in both present and future treatments (Table 1). Total alkalinity was measured weekly via open-cell potentiometric titration. Carbonate parameters within each tank were calculated in the CO_2_SYS program using measured parameters of pH, TA, temperature, and salinity, with the carbonate dissociation constants of Mehrbach et al. [44] as refit by Dickson & Millero [45].

**Table 1:** Average (±SE) calculated carbonate chemistry parameters from the measured parameters of pH, total alkalinity (TA), temperature, and salinity (n = 6).

Experiment	Treatment	Salinity	Temp (°C)	TA (μmol kg^*-1*^)	pH_*NBS*_	pCO_*2*_ (μatm)	CO_*2*_ (μmol kg^*-1*^)	HCO_*3*_^*-*^ (μmol kg^*-1*^)	*Ω*_*ca*_	*Ω*_*ar*_
*P. astreoides* vs. *M. cavernosa*	Present	36.18	28.60	2340.49	8.18	373.75	9.55	1639.80	5.68	3.80
		±0.15	±0.07	±33.12	±0.02	±21.82	±0.56	±90.40	±0.40	±0.26
	Future	35.62	30.24	2474.24	7.89	1083.4	26.68	2125.99	3.49	2.35
		±0.22	±0.09	±50.51	±0.01	±52.61	±1.21	±47.05	±0.07	±0.05
	p-values	p = 0.032	p<0.001	p = 0.094	p<0.001	p<0.001	p<0.001	p = 0.001	p = 0.001	p = 0.001
*P. astreoides* vs. *O. faveolata*	Present	36.30	28.47	2137.73	8.17	460.70	11.93	1655.87	4.60	3.07
		±0.17	±0.05	±23.87	±0.01	±14.93	±0.40	±21.69	±0.11	±0.07
	Future	36.59	30.28	2352.72	7.86	1107.99	27.31	2025.81	3.21	2.16
		±0.39	±0.11	±25.56	±0.01	±47.53	±1.12	±20.02	±0.15	±0.10
	p-values	p = 0.660	p<0.001	p = 0.002	p<0.001	p<0.001	p<0.001	p<0.001	p<0.001	p<0.001

Coral fragments were randomly assigned to treatments and tanks. Two pairs of fragments (one from each species) were positioned as follows: i) one pair was positioned ~10 cm apart from each other to prevent any interactions via mesenterial filaments or sweeper tentacles [36] and ii) a second pair was placed similarly but was then moved into contact after the acclimation period discussed below. This design resulted in the use of 24 fragments per species. Given the limited number of coral individuals (3) per species that were available for this experiment, this design could have confounded tank and individual level effects, but the random allocation of coral fragments to tanks should have minimized this. Coral fragments acclimated to tank conditions for 7 days without competition, but after the 7-day acclimation contact was initiated for one of the pairs in each tank. One fragment of *M*. *cavernosa* was placed in direct contact with one fragment of *P*. *astreoides*, and this treatment was maintained for 7 days. The area of direct contact was 6–7 cm in the area of contact, and living surfaces were placed in contact with polyps oriented toward the surface of the other coral. The remaining fragments of each coral (no-contact controls) in each tank were positioned 10 cm away from the contact corals and from each other.

Endosymbiont photosynthetic efficiency was monitored using PAM fluorometry, one method for assessing coral health [46] and for quantifying coral bleaching [47], to determine maximum quantum yield (F_v_/F_m_−a measure of photosynthetic efficiency) every other day over the course of the experiment. PAM fluorometry has been used previously in algal-coral competition studies and has shown that reduced F_v_/F_m_ commonly leads to coral bleaching and often death [48, 49]. Additionally, reduced F_v_/F_m_ is also associated with rapid changes in coral immune responses, protein degradation, and changes in catalytic and metabolic activity, which can all lead to apoptosis and necrosis of coral tissues [50, 51]. Corals were dark-adapted for one hour prior to measuring F_v_/F_m_, and readings were taken between 1100 and 1300 hours each day. For each coral in direct contact, a single F_v_/F_m_ measurement was taken at the area of contact with the other species and two measurements were taken approximately 3 cm away from the area of contact, but on this same fragment. The average of these two measurements was designated as the average F_v_/F_m_ for the “no-contact” portions of the coral, and the difference between the direct contact and the average “no-contact” F_v_/F_m_ on that same fragment was compared. For the “no-contact control” corals, three F_v_/F_m_ measurements were taken haphazardly over the coral to get an average for that individual. This average value was compared to the aforementioned “no-contact” location F_v_/F_m_ for the coral in contact with the other species. When coral lesions formed (this happened only for corals in contact), F_v_/F_m_ thereafter increased to values outside of the average readings for no-contact corals in this experiment. This appeared to be due to rapid colonization of the exposed coral skeleton by diatoms, cyanobacteria, or other epilithic algae (as also noted by McCook et al. 2001 [24]). Areas with a visible lesion (confirmed with photos and a zero or near zero F_v_/F_m_ prior to epilithic algal growth) were recorded as a “zero” F_v_/F_m_−even following colonization by epilithic algae.

The combined effects of OA and warming on competition were evaluated by comparing differences in endosymbiont photosynthetic efficiency between points of direct coral-coral contact and points on the same piece of coral that were 3 cm distant from contact with the other species and evaluating these patterns across the two environment scenarios (present vs future). The effects of competition and environment on coral health beyond the areas of direct contact were evaluated by comparing photosynthetic efficiency values taken from areas of the competing corals that were 3 cm away from the direct contact area and comparing these values to values from corals in the same tank but not in contact with a competitor (the “no-contact” controls).

Lesion presence and size were recorded through daily photos (including a scale) using a Nikon Coolpix W300 camera to evaluate the effects of competition and OA/warming on coral tissue health. Lesions formed first via visible discoloration of coral tissue followed by tissue mortality and tissue loss. Distinctions were made between areas of the lesion that were discolored (i.e. areas where the color differed from other parts considered healthy) and areas with exposed coral skeleton and tissue mortality. Total lesion size was quantified as the combined size of the discolored area and the area with tissue mortality. The total area of tissue mortality was also quantified. After the first 2–3 days of lesion formation, lesion size stabilized, so the size of the areas that were discolored and/or contained dead tissue were statistically evaluated only at day 7 using ImageJ software.

### Experiment two: *Porites astreoides* vs *Orbicella faveolata*

Because coral competitive outcomes can change over time as sweeper tentacles form in response to competition [31, 37] and because we wanted to evaluate among-species differences in competitive outcomes, we conducted a follow-up experiment using *P*. *astreoides* and *O*. *faveolata*. This experiment mimicked the previous experiment’s design with minor variations (Table 1). Three colonies of each species acclimated in the original raceways for an additional period of three weeks while we conducted the first experiment. As with the first experiment, they were then cut into 8 fragments and acclimated for 48 hours before being transferred into the 12 tank experimental system. After acclimating to the experimental system for 7 days, interspecific coral interactions were initiated and monitored over a 14-day period (7 days longer than the previous experiment) to determine whether the effects of future conditions altered the outcomes of coral-coral competition. Once again, daily photos recorded lesion presence and changes in size. Photos were analyzed from day 7 and from day 14, at the end of the experiment. F_v_/F_m_ was recorded every other day as in the first experiment.

### Statistical analyses

All analyses were conducted in R version 3.4.3 using the car 2.1–5 [52], lmPerm 2.1.0 [53], and multcomp 1.0 packages [54]. Data were evaluated for normality and equality of variance prior to analyses using the Bartlett’s test and Q-Q plots to analyze plots of residuals. Two way-permutation ANOVAs were used when data did not meet the assumptions of equality of variance and transforming the data failed to meet these assumptions using climate (present vs. future) and contact (contact vs no-contact) as fixed effects. The effect of individual tanks on the data was analyzed using a one-way ANOVA and, lacking significance, was removed from the analysis. All other analyses were conducted using two-way ANOVAs with climate and contact area as fixed effects. Post-hoc tests were completed using one-way ANOVAs with the Bonferroni correction to evaluate differences among treatments. Contrasts of physical parameters (temperature, OA, salinity, etc.) between treatments were conducted by averaging the many measures for each tank across time into one mean for each tank (i.e., using tanks as independent replicates) and comparing the six present treatment tanks to the six future treatment tanks via t-tests.

## Results

**Fig 1:**
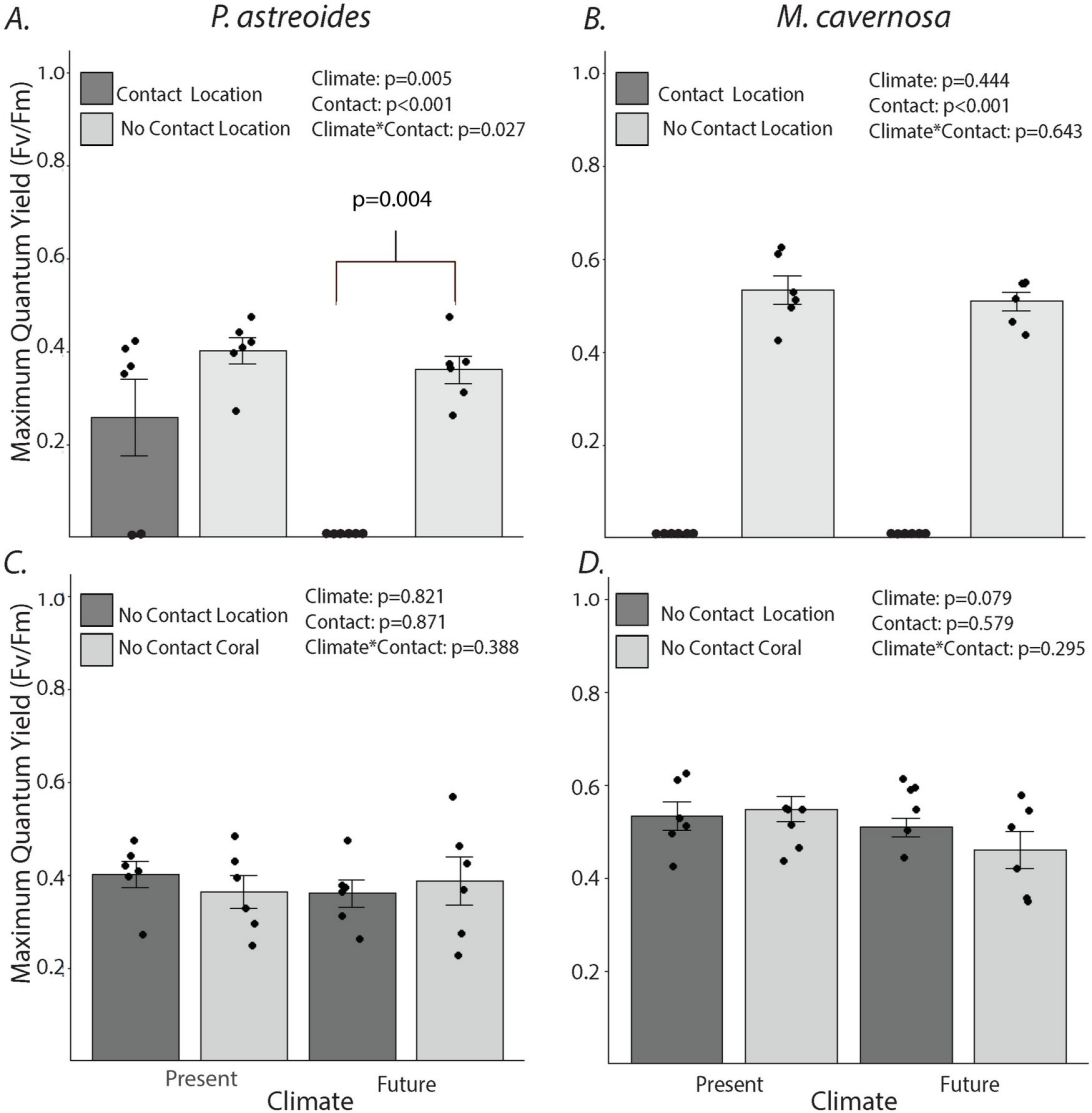
Effects of climate and coral-coral competition on endosymbiont photosynthetic efficiency (F_v_/F_m_). Maximum quantum yield (means ± SE) for (A) *Porites astreoides* in contact with *Montastraea cavernosa* at the location of direct contact (Contact Location) vs. 3 cm away from area of direct contact (No Contact Location) and (B) *M*. *cavernosa* in contact with *P*. *astreoides* at the Contact Location vs. No Contact Location. Maximum quantum yield values (means ± SE) for (C) *P*. *astreoides* in contact with *M*. *cavernosa* at points 3 cm away from site of direct contact (No Contact Location) vs. a control *P*. *astreoides* fragment not in direct contact with *M*. *cavernosa* (No Contact Coral) and (D) *M*. *cavernosa* in contact with *P*. *astreoides* at points 3 cm away from direct contact (No Contact Location) vs. a control *M*. *cavernosa* fragment not in direct contact with *P*. *astreoides* (No Contact Coral). Analyses by two-way ANOVA comparing contact and climate for each coral species. Dots show individual data points. The p-value contrasting values between “Contact Location” and “No Contact Location” under future conditions is from one-way ANOVA (N = 6).

**Fig 2:**
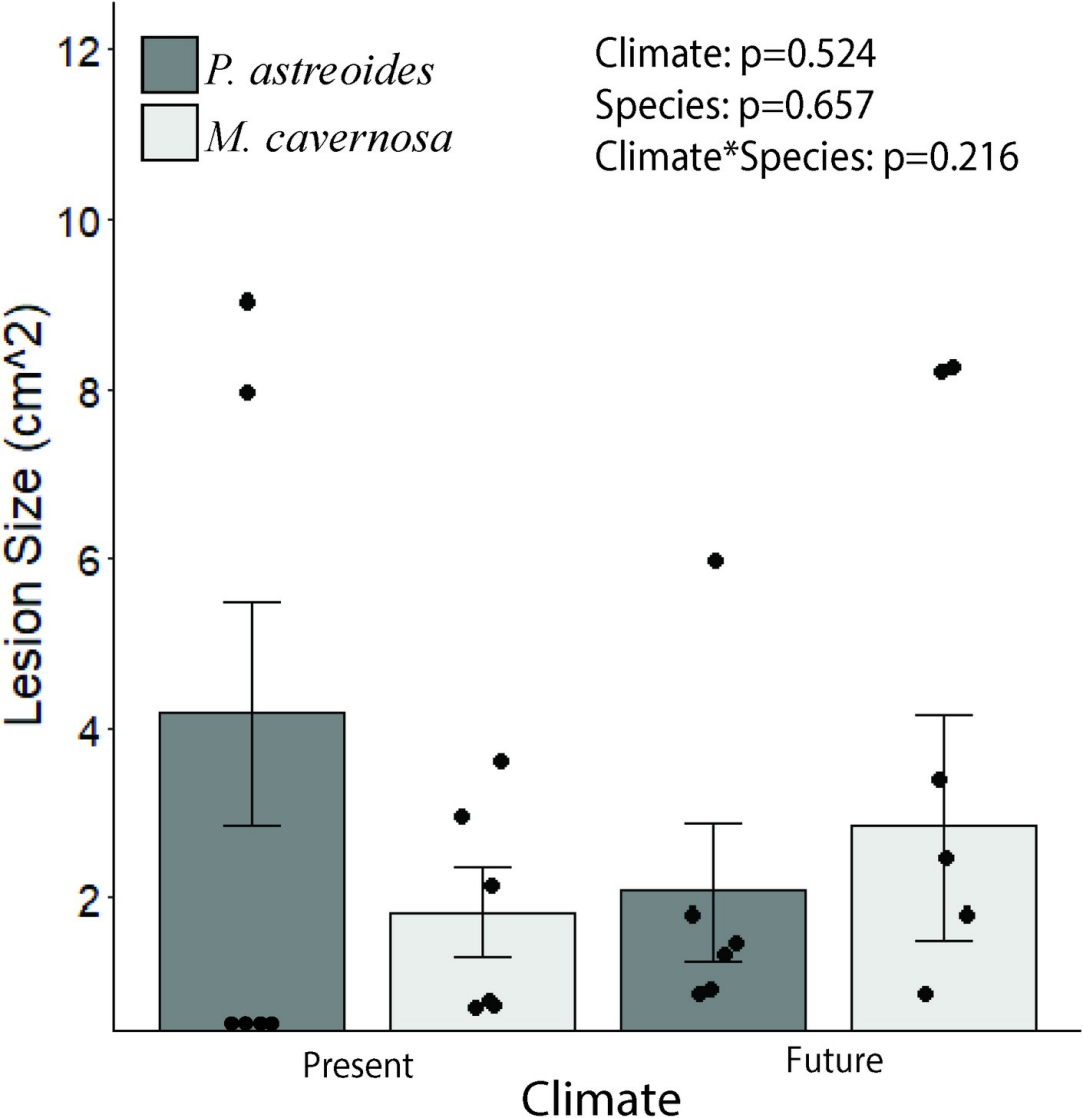
Lesion size (means ±SE) for *Porites astreoides* and *Montastraea cavernosa* at Day 7. Only corals in direct contact were included in the analysis. Evaluated via a two-way permutational ANOVA comparing climate and species. Dots represent individual data points. N = 6.

Elevated temperature and CO_2_ significantly changed competitive interactions between *M*. *cavernosa* and *P*. *astreoides* (Fig 1A, climate x contact, p = 0.027). Under present climate conditions, contact with *M*. *cavernosa* for 7 days reduced *P*. *astreoides* F_v_/F_m_ by 37.5%, but under future temperature and OA, contact reduced F_v_/F_m_ by 100%. For *M*. *cavernosa*, direct contact with *P*. *astreoides* reduced F_v_/F_m_ at the point of contact by 100%, regardless of climate conditions (Fig 1B, climate x contact, p = 0.643). Neither climate condition, nor competition, affected F_v_/F_m_ in non-contact areas (Fig 1C & 1D). Temperature and OA also did not significantly impact the size of the lesion formed at the point of contact for either coral species (Fig 2).

**Fig 3:**
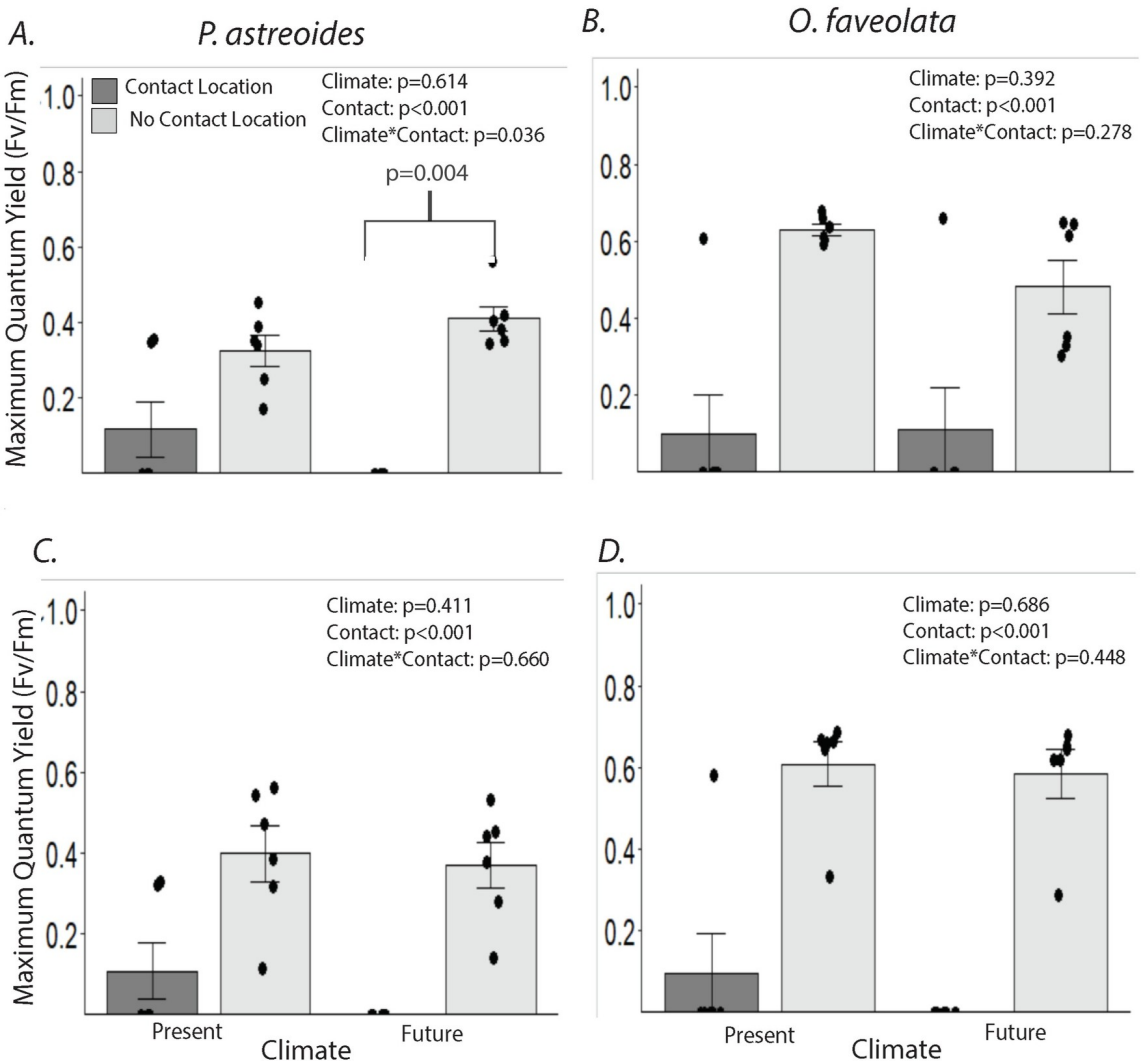
Effects of climate and coral-coral competition on endosymbiont photosynthetic efficiency (F_v_/F_m_). Maximum quantum yield values (means ± SE) for (A) *Porites astreoides* in contact with *Orbicella faveolata* at the location of direct contact (Contact Location) vs. 3 cm away from area of direct contact (No Contact Location) at day 7, (B) *O*. *faveolata* in contact with *P*. *astreoides* at the Contact Location vs. No Contact Location at day 7, (C) *P*. *astreoides* in contact with *O*. *faveolata* at the Contact Location vs. No Contact Location at day 14, and (D) *O*. *faveolata* in contact with *P*. *astreoides* at the Contact Location vs. No Contact Location at day 14. Analyzed with two-way ANOVAs comparing contact area and climate condition. Dots represent individual data points. The p-value contrasting values between “Contact Location” and “No Contact Location” under future conditions is from one-way ANOVA N = 6.

**Fig 4:**
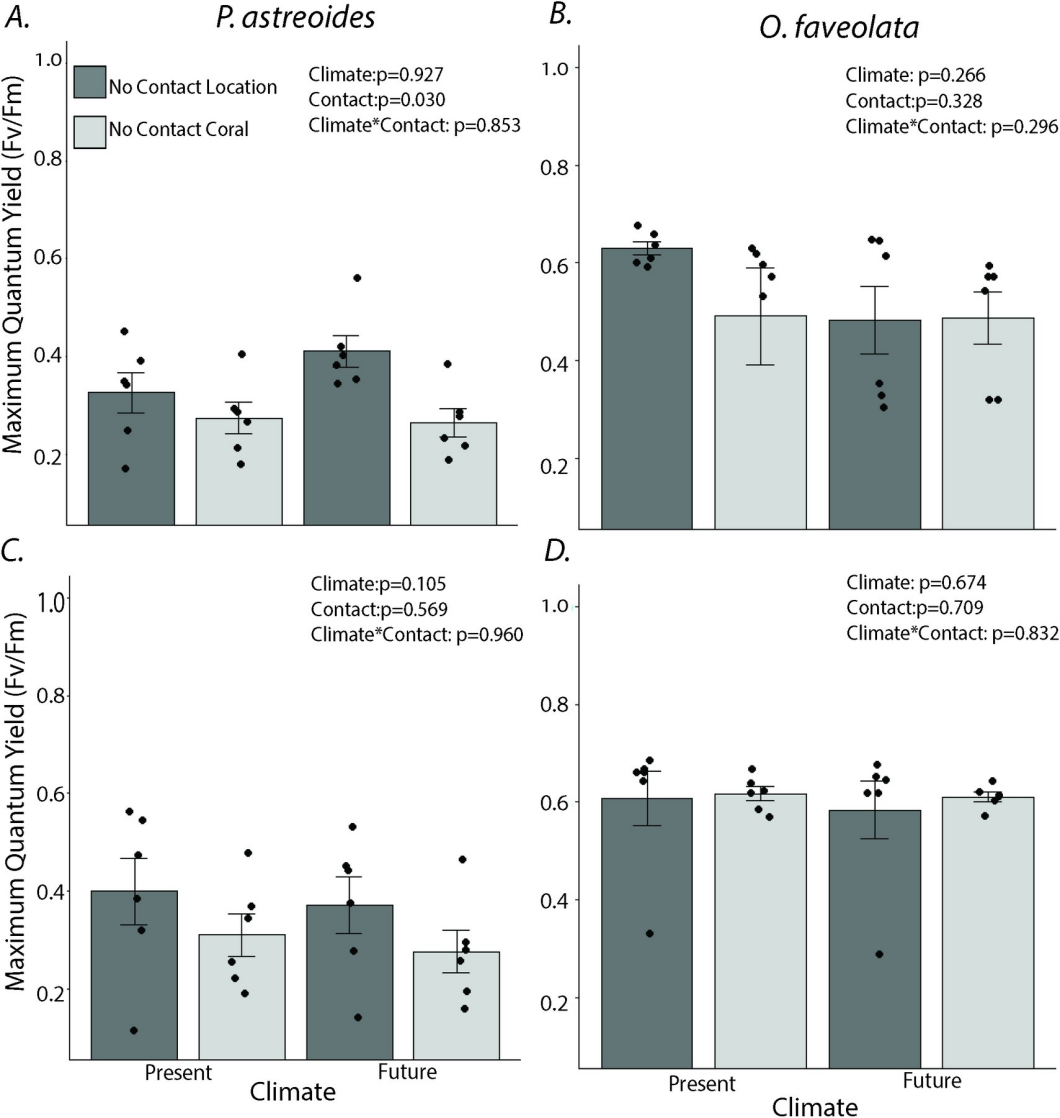
Effects of climate and coral-coral competition on maximum quantum yield (means ±SE) beyond areas of direct contact. Maximum quantum yield (means ± SE) for (A) *Porites astreoides* in contact with *Orbicella faveolata*; measurement taken at the point 3 cm away from direct contact (No Contact Location) vs. a control *P*. *astreoides* fragment not in direct contact with *O*. *faveolata* (No Contact Coral) at day 7, (B) The same contrast but for *O*. *faveolata* in contact with *P*. *astreoides*, (C) *P*. *astreoides* in contact with *O*. *faveolata*: measurement taken at the point 3 cm away from direct contact (No Contact Location) vs. a control *P*. *astreoides* fragment not in direct contact with *O*. *faveolata* (No Contact Coral) at day 14, and (D) The same contrast, but for *O*. *faveolata* in contact with *P*. *astreoides*. Analyses by two-way ANOVA comparing contact and climate condition for each coral species. Dots show individual data points. N = 6.

Areas of *P*. *astreoides* in contact with *O*. *faveolata* exhibited a 62.5% reduction in F_v_/F_m_ under present conditions, but reductions of 100% under future OA and warming conditions at day 7 (Fig 3A, climate x contact, p = 0.036). For *O*. *faveolata*, contact with *P*. *astreoides* reduced F_v_/F_m_ by 77–84% under both climate conditions (contact: p<0.001), and effects did not vary as a function of OA and temperature (Fig 3B; climate x contact: p = 0.278). At day 14, the patterns for *P*. *astreoides* were similar to those on day 7. Contact reduced *P*. *astreoides* F_v_/F_m_ by 45–62% (contact: p<0.001), but there was no longer a significant contact x climate interaction at day 14 (Fig 3C, p = 0.660). For *O*. *faveolata*, patterns on day 7 persisted through day 14; competition reduced F_v_/F_m_ by 84–100% (Fig 3D p<0.001), with no significant difference between climate conditions. As with the *P*. *astreoides-M*. *cavernosa* interaction, negative effects of competition on F_v_/F_m_ were restricted to areas of direct contact. For *P*. *astreoides*, areas not in direct contact with *O*. *faveolata* exhibited significantly higher F_v_/F_m_ than the “no-contact controls” on day 7, but by day 14, this pattern was no longer significant (Fig 4).

**Fig 5:**
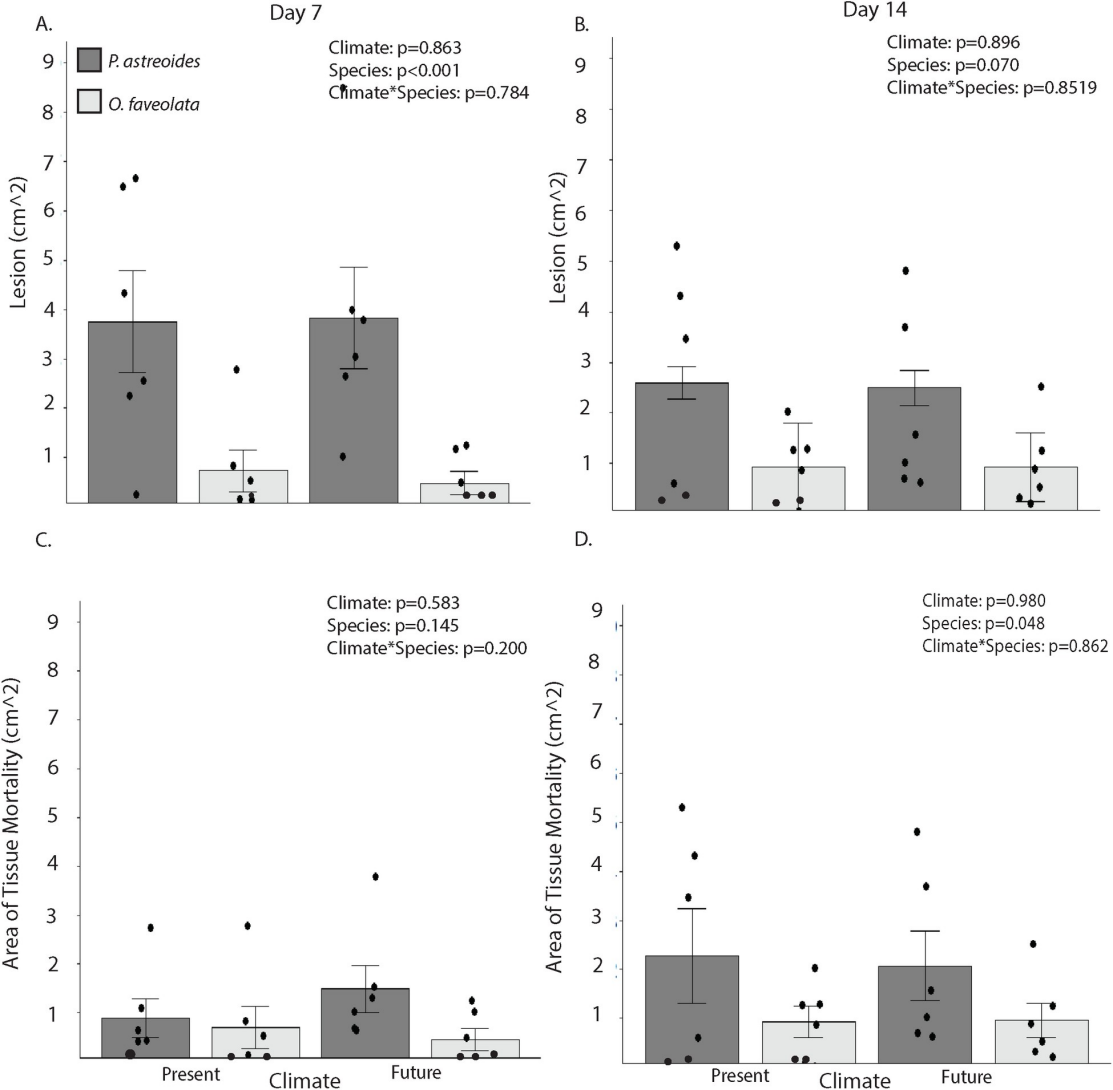
Lesion size and tissue mortality at Day 7 and Day 14. Lesion size (means ±SE) (A and B) and Area of Tissue mortality (means ±SE) (C and D) for each coral in contact with the other at day 7 (A and C) and day 14 (B and D). Lesion area is the area of each coral that exhibited discolored coral tissue plus the area where coral tissues have died and been lost. Area of tissue mortality corresponds to areas of bare skeleton with no living tissue. Analyzed with two-way Permutational ANOVA comparing species and climate. Dots represent individual data points. N = 6.

As with the interaction between *P*. *astreoides* and *M*. *cavernosa*, lesion sizes of *P*. *astreoides* and *O*. *faveolata* were unaffected by temperature and OA (Fig 5). However, *P*. *astreoides* developed discoloration areas indicative of lesion formation that were 4x larger than those of *O*. *faveolata* at day 7 (Fig 5A, p<0.001) although there were no significant differences in the portions of the coral with bare skeleton (Fig 5C, p = 0.45). By day 14, the overall size of the discoloration area was no longer significantly different between the two coral species (Fig 5B, p = 0.070). However, the area with complete loss of coral tissue was 133% larger on *P*. *astreoides* than *O*. *faveolata* (Fig 5D, p = 0.048).

## Discussion

Most experiments directly measuring coral-coral interactions were conducted decades ago when coral reefs were healthier, coral cover was greater, and coral-coral contact was more frequent. Those studies found that coral-coral interactions were hierarchical [29, 30], but could sometimes reverse over longer periods of contact [31]. Here we show that climate change can impact the speed or severity of coral-coral competition for some species that are among the most common corals remaining on Caribbean reefs [42]. Competition with *P*. *astreoides* negatively impacted *M*. *cavernosa* and *O*. *faveolata* in areas of direct contact regardless of climate treatment, however *P*. *astreoides* became more negatively impacted by contact with the other two corals in areas of direct contact under predicted levels of OA and warming.

Many experiments evaluating the effect of OA on coral reef competitive interactions have found that high CO_2_ either enhances the susceptibility of the weaker competitor to the stronger competitor (e.g. high CO_2_ enhancing algal competition over corals [55]), reverses the competitive dynamics (e.g. in damselfish [56]), affects intraspecific competition more strongly than interspecific competition [41], or may not directly affect interspecific competition [40]. Unlike these results, our data suggest that OA and warming increased the susceptibility of the stronger competitor, *P*. *astreoides*, to the weaker competitors, *M*. *cavernosa* and *O*. *faveolata*, in areas of direct contact at day 7 without impacting the negative effects that *P*. *astreoides* had on the weaker competitors. Outside of direct contact areas, none of the corals exhibited evidence of reductions in photosynthetic efficiency from competitive interactions or OA and temperature stress at day 7 (Figs 1C, 4A and 4B). The mechanism altering the susceptibility of *P*. *astreoides* to competition with *O*. *faveolata* and *M*. *cavernosa* in areas of direct contact under OA and warming is unclear. It is possible that the change in susceptibility could have been exacerbated by differences in salinity between present and future treatments. While corals are known to have a limited tolerance for changes in salinity [57], the small differences in salinity between treatments (<0.6 ppt) were unlikely to have affected coral performance. Previous research evaluating effects of salinity on *Orbicella faveolata* larvae (which we assume are less robust than adults) found that differences of 4 ppt (36 vs. 32 ppt) affected larval survival but that a difference of 2 psu (36 vs 34 ppt) did not [58]. The change in the susceptibility of *P*. *astreoides* to the other two corals is more likely to be due to oxidative stress, which is often a precursor to coral bleaching [59–61]. changes in the microbiome [62], altered chemical defenses [63], faster mesenterial filament production [40], or other mechanisms. Regardless of the factor, these results suggest that the OA and warming may alter coral-coral interactions among some of the most common species remaining on degraded Caribbean reefs.

While both experiments demonstrated an effect of OA and warming on coral-coral interactions at day 7, the second experiment ran for an extra 7 days to evaluate if results changed over time. Other experiments have suggested that ecological interactions can outweigh the negative effects of OA and warming over longer time scales [40]. As with these studies, OA and warming no longer significantly affected photosynthetic efficiency in areas of direct contact at day 14 (Fig 3). Increased variation of photosynthetic efficiencies in the no contact locations for both present and future treatments (Fig 3A and Fig 3C) corresponded with an increase in the size of the tissue necrosis area from day 7 to day 14 for *P*. *astreoides* suggestive of continued competition (Fig 5C and 5D). These results mirror other findings suggesting that OA can speed the production of mesenterial filaments while not affecting the final outcome of competition [40].

Across both experiments, areas of discoloration and/or tissue mortality formed in response to competition, but the sizes of these areas were unaffected by OA and warming. In the first experiment, damage did not expand beyond the areas of direct contact, and there was no significant difference in the size of the lesion area between the two coral species (Fig 2). In the second experiment, *P*. *astreoides* developed areas of discoloration twice the size of those formed by *O*. *faveolata*, and these extended beyond the areas of direct contact by day 7 (Fig 5). This may be evidence of the formation of sweeper tentacles known to form on some corals [31, 36], although the evidence of such formation was not visually obvious in this study. The area of discoloration on *P*. *astreoides* shrank 41% between days 7 and 14, leaving tissue mortality and bare skeleton in the areas of direct contact with recovered tissue outside areas of direct contact. Some studies have suggested that environmental conditions can play a role in coral tissue recovery [64, 65], however they focused more on strong variations between environmental field conditions without evaluating the specific conditions responsible. In this study, there was no evidence that returned tissue coloration (suggestive of tissue recovery) was dependent on present or future warming conditions supporting one other study that found tissue recovery of *Porites spp*. to be unaffected by OA [66].

Across the two experiments, OA and warming only affected competition in areas of direct contact. Areas of the coral not in direct contact and the no-contact control corals did not demonstrate evidence of loss of coloration or differences in F_v_/F_m_. It is possible that these results may have changed if the experiment had run over a longer period of time. Regardless, our results demonstrate that ecological interactions can be affected by environmental stress before common signs of physiological stress (e.g. loss of coloration and, potentially, subsequent bleaching) are visible. It may be that a trade-off exists between an ability of a coral to respond to competition and its ability to withstand OA and warming as a coral re-allocates resources away from fending off competition to withstanding environmental stress. Our results highlight that predicting the effects of OA and warming on ecological interactions may require direct tests under predicted future conditions and that predicting outcomes based on physiology alone may be challenging.

The goal of this study was to evaluate how the dual factors of OA and warming affected coral-coral interactions among some of the common mounding corals remaining on Caribbean reefs. While it is not possible to make conclusions about the relative importance of the two environmental factors in this study, multiple studies have documented the immediate effect that temperature stress can have on corals–particularly during recent mass bleaching events [4]. Follow-up studies considering the relative importance of these two factors and evaluating the generality of effects would be useful. Of particular interest would be enhanced understanding of the potential trade-off between environmental stress response and competition and the effect of any potential for these corals to acclimate and/or adapt to effects of a changing climate on ecological interactions. Some corals can increase their resilience to environmental stress over time through changes in gene expression [67]. Differential ability to acclimate to OA and/or warming would likely impact competitive interactions and should be an area of focus.

Competitive interactions between *P*. *astreoides* and *M*. *cavernosa* or *O*. *faveolata* suggest a competitive hierarchy such as those found in earlier coral competitive interaction studies [29, 30]. However, similar to the model evaluated by Horwitz et al. [41], our findings also suggest that competitive hierarchies may change or become more variable under OA and warming. If these changes occur in nature as oceans warm and acidify, this may establish a new competitive relationship among remaining corals on reefs in the Caribbean. *P*. *astreoides* is among the most abundant corals in the Florida Keys and is more abundant than either *O*. *faveolata* or *M*. *cavernosa* [42, 68]. It was also relatively resistant to *O*. *faveolata* and *M*. *cavernosa* contact under present conditions; however, its advantage is compromised as warming and acidification increase. *O*. *faveolata* is the least abundant of the three species [42], but it and *P*. *astreoides* had similar effects on each other’s F_v_/F_m_ (Fig 3), and *O*. *faveolata* caused larger lesions on *P*. *astreoides* than *P*. *astreoides* did on *O*. *faveolata* (Fig 5). These results suggest that the current dominant coral on Caribbean reefs may become more compromised under predicted future conditions of OA and warming.

Coral-coral interactions are less common on modern, degraded reefs; however, they still occur in areas where coral persists at higher cover [41]. Understanding and predicting the outcome of these interactions may be of increasing relevance as rising temperatures and increasing OA alter community dynamics of future reefs.
